# The Motility of Mouse Spermatozoa Changes Differentially After 30-Minute Exposure Under Simulating Weightlessness and Hypergravity

**DOI:** 10.3390/ijms252413561

**Published:** 2024-12-18

**Authors:** Irina V. Ogneva, Yulia S. Zhdankina, Ksenia K. Gogichaeva, Artyom A. Malkov, Nikolay S. Biryukov

**Affiliations:** 1 Cell Biophysics Laboratory, State Scientific Center of the Russian Federation Institute of Biomedical Problems of the Russian Academy of Sciences, 76a, Khoroshevskoyoe Shosse, 123007 Moscow, Russia; juliaszd@yandex.ru (Y.S.Z.); xeniagogichaeva@gmail.com (K.K.G.); malkov.aa.2003@yandex.ru (A.A.M.); biryukovns@gmail.com (N.S.B.); 2 Medical and Biological Physics Department, I.M. Sechenov First Moscow State Medical University, 8-2 Trubetskaya Street, 119991 Moscow, Russia; 3 Yu.A. Gagarin Research and Test Cosmonaut Training Center, 141160 Star City, Moscow Region, Russia

**Keywords:** cell mechanosensitivity, microgravity, hypergravity, spermatozoa, cytoskeleton

## Abstract

Research into the mechanisms by which gravity influences spermatozoa has implications for maintaining the species in deep space exploration and may provide new approaches to reproductive technologies on Earth. Changes in the speed of mouse spermatozoa after 30 min exposure to simulated weightlessness (by 3D-clinostat) and 2 g hypergravity (by centrifugation) were studied using inhibitory analysis. Simulated microgravity after 30 min led to an increase in the speed of spermatozoa and against the background of an increase in the relative calcium content in the cytoplasm. This effect was prevented by the introduction of 6-(dimethylamino) purine, wortmannin, and calyculin A. Hypergravity led to a decrease in the speed of spermatozoa movement, which was prevented by sodium orthovanadate and calyculin A. At the same time, under microgravity conditions, there was a redistribution of proteins forming microfilament bundles between the membrane and cytoplasmic compartments and under hypergravity conditions—proteins forming networks. The obtained results indicate that even a short exposure of spermatozoa to altered gravity leads to the launch of mechanotransduction pathways in them and a change in motility.

## 1. Introduction

The future of humanity in outer space will most likely be associated with the exploration of the Moon, Mars, and more distant bodies of the Solar System. At the same time, the increase in the duration of space flights will eventually raise the question of the implementation of the reproductive function of space explorers and, perhaps, the overall maintenance of the species. And, if earlier in space medicine, the influence of space flight factors on the reproductive system attracted little attention from researchers, then recently the number of studies has increased [1,2,3].

Weightlessness, real or simulated, leads to a decrease in sperm production [4,5,6] and motility [7,8] in mammals in vivo, including through changes in the differentiation potential of progenitor cells [9], the cytoskeleton, and gene expression in the tissues of the testes and duct deferens [8,10].

In vitro experiments using native (non-frozen) spermatozoa, as well as in vivo, have shown that after a parabolic flight, motility decreases [11]. However, after 6 min of suborbital flight, the speed of bull spermatozoa was higher than in the control [12]. Although, a longer exposure, for 6 h, led to a decrease in the speed of mouse spermatozoa due to changes in protein kinase activity [13,14]. Interestingly, in lower animals, sperm motility increases under these conditions. Thus, sea urchin spermatozoa under spaceflight conditions demonstrate an increase in motor activity due to a change in the phosphorylation level of motor proteins [15]. Spermatozoa of the fruit fly *Drosophila melanogaster* also move faster under simulated microgravity conditions [14]. Although it should be noted that after the in vivo experiment—spaceflight—the speed of *Drosophila* spermatozoa decreased, the effect was due to overloads and the early period of re-adaptation to the Earth’s gravity [16].

Nevertheless, the development of technologies in reproductive medicine provides opportunities for preserving genetic material in an intact state; in particular, cryopreservation of spermatozoa is possible. Thus, frozen spermatozoa of mice after long-term storage on board the ISS were returned to Earth and used to obtain offspring. Despite the existing DNA damage, fertile offspring were obtained, which had virtually no differences from the control group [17]. Frozen samples of human spermatozoa were studied after a parabolic flight, which included 20 parabolas. The authors showed that none of the spermogram parameters changed significantly after the experiment relative to the control group [18]. However, it should be noted that these studies of preserved spermatozoa after exposure were carried out on Earth under normal gravity. If deconservation occurs in altered gravity, even a short stay in these conditions, for example, when performing manipulations during artificial fertilization, can lead to various changes. For example, for *Drosophila melanogaster* oocytes, we found different responses to simulated micro- and hypergravity after 30 min of exposure, although they leveled off after longer, up to 6 h, cultivation [19].

Given the possibility of short exposure to altered gravity, we decided to analyze the motility of mouse spermatozoa after 30 min of exposure to simulated weightlessness and hypergravity at 2 g. In our previous studies, we showed that mammalian sperm motility in microgravity can be altered by the action of protein kinases, with an unchanged content of proteins that form the tubulin–dynein motility system [14]. Therefore, in this work, the main focus was on identifying possible targets using specific inhibitors. In addition, given the role of calcium in sperm motility, we assessed its relative content and identified the main cytoskeletal proteins.

## 2. Results

### 2.1. Speed of Sperm Movement

**Figure 1:**
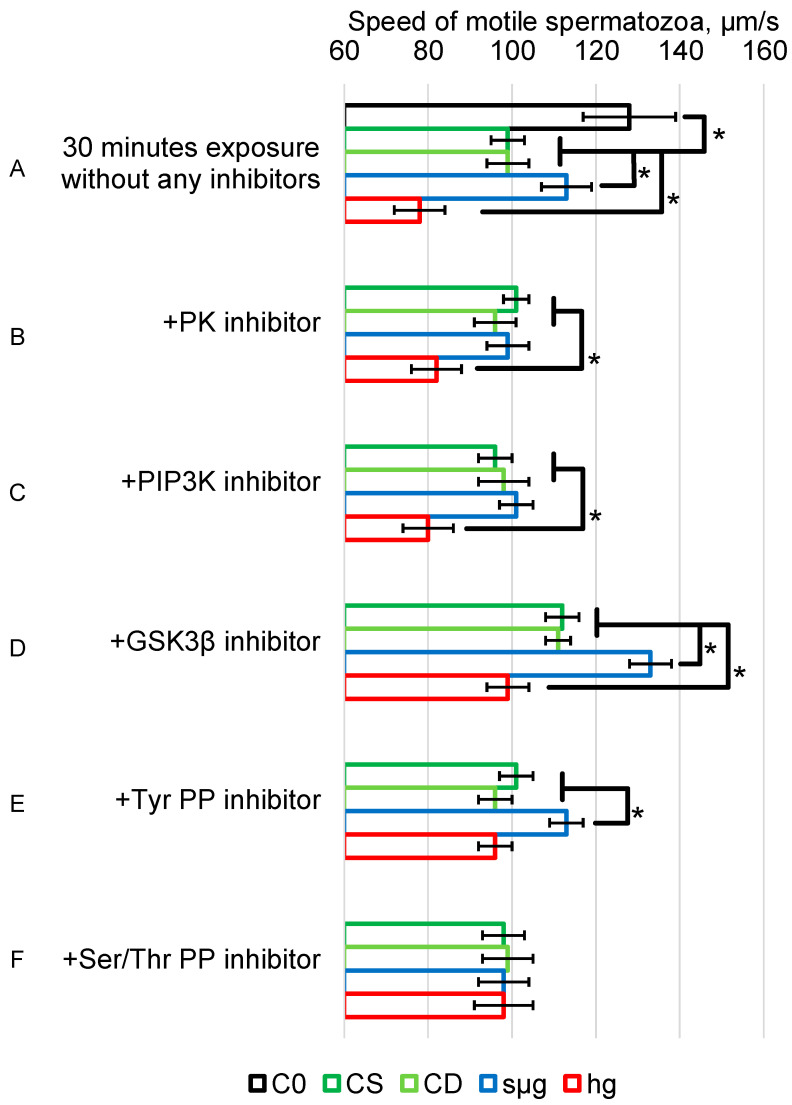
Speed of mouse spermatozoa after 30 min exposure to simulated micro- and hypergravity. Study groups: C0—“zero-control”, parameters were assessed immediately after sperm collection, CS—static control—intact spermatozoa after 30 min of exposure without any impacts (dark green), CD—dynamic control—spermatozoa after 30 min of exposure on shaker with similar velocity of rotation in one plane (light green), sµg—simulated microgravity during 30 min, created by 3D-clinostat (blue), hg—hypergravity at 2 g level during 30 min, created by centrifugation (red). Values for CS and CD did not differ from each other. *—*p* < 0.05 in comparison to CS. (**A**) Exposure was carried out in a medium for spermatozoa without the addition of any agents. (**B**) Exposure was carried out in a medium with the addition of a broad-spectrum protein kinase inhibitor 0.5 mM 6-(dimethylamino) purine. (**C**) The exposure medium contained 20 μM wortmannin to inhibit phosphatidylinositol-3-kinase (PI3K). (**D**) The medium was supplemented with 1 μM AR-A014418, an inhibitor of glycogen synthase kinase 3 (GSK3). (**E**) The exposure medium contained 200 μM sodium orthovanadate to inhibit tyrosine phosphatase (Tyr PP). (**F**) Spermatozoa were exposed in the medium containing 20 nM calyculin A, which inhibits serine/threonine phosphatase (Ser/Thr PP). Data are presented as mean ± SD of three independent experiments. In each independent experiment, at least 50 cells were assessed for motility in each study group. Motility in each study group was compared with the corresponding CS group.

The sperm motility after 30 min of exposure in the CS control group (control static—intact spermatozoa after 30 min of exposure without any impacts) was lower than the initial one in the C0 (control at 0 min of exposure—intact spermatozoa just after collection) group by 23% (*p* < 0.05) (99 ± 4 µm/s vs. 128 ± 11 µm/s) (Figure 1A). The sperm motility in the static (CS) and dynamic (CD) control groups did not differ between each other in all experimental series (Figure 1A–F).

In the medium without the addition of inhibitors, the sperm motility under simulated microgravity (group sµg, simulated microgravity was created by a 3D-clinostat) was higher than that in the CS group by 14% (*p* < 0.05) (113 ± 6 µm/s vs. 99 ± 4 µm/s). In contrast, in the hypergravity (hg) group (created by centrifugation), the speed was lower by 21% (*p* < 0.05) compared with the CS group (78 ± 6 µm/s vs. 99 ± 4 µm/s) (Figure 1A).

When sperm were exposed with the broad-spectrum kinase inhibitor, 6-DMAP, the speed in the sµg group was not different from the corresponding control; but, in the hg group, it remained 18% lower (*p* < 0.05) (82 ± 6 µm/s vs. 101 ± 3 µm/s) (Figure 1B).

Incubation with a specific PI3K inhibitor, wortmannin, produced a similar effect. In this case, there were also no differences between sperm speed in the simulated microgravity group and the control; but, in the hypergravity group, the speed remained 20% lower than the corresponding control (*p* < 0.05) (80 ± 6 µm/s vs. 96 ± 4 µm/s) (Figure 1C).

It should be noted that 30 min incubation of spermatozoa from the control groups CS and CD with 6-DMAP and wortmannin did not result in a change in motility. However, incubation with the GSK3 inhibitor, AR-A014418, stimulated sperm motility in all study groups compared to that without the addition of any agents (Figure 1D vs. Figure 1A). Thus, in the CS group, against the background of the GSK3 inhibitor, the speed was 112 ± 4 µm/s (vs. 99 ± 3 µm/s in a pure medium), CD—111 ± 3 µm/s (vs. 99 ± 5 µm/s), sµg—133 ± 5 µm/s (vs. 113 ± 6 µm/s), and hg—99 ± 5 µm/s (vs. 78 ± 6 µm/s).

Incubation of spermatozoa of the CS and CD groups with both PP inhibitors did not change their motility. With the addition of the Tyr PP inhibitor, sodium orthovanadate, the speed in the sµg group remained higher than the control by 15% (*p* < 0.05) (113 ± 4 µm/s vs. 101 ± 4 µm/s) but did not differ from the control in the hg group (Figure 1E). Addition of the Ser/Thr PP inhibitor, calyculin A, to the incubation medium prevented changes in the speed of spermatozoa under simulated weightlessness and hypergravity (Figure 1F).

### 2.2. Calcium Relative Content

**Figure 2:**
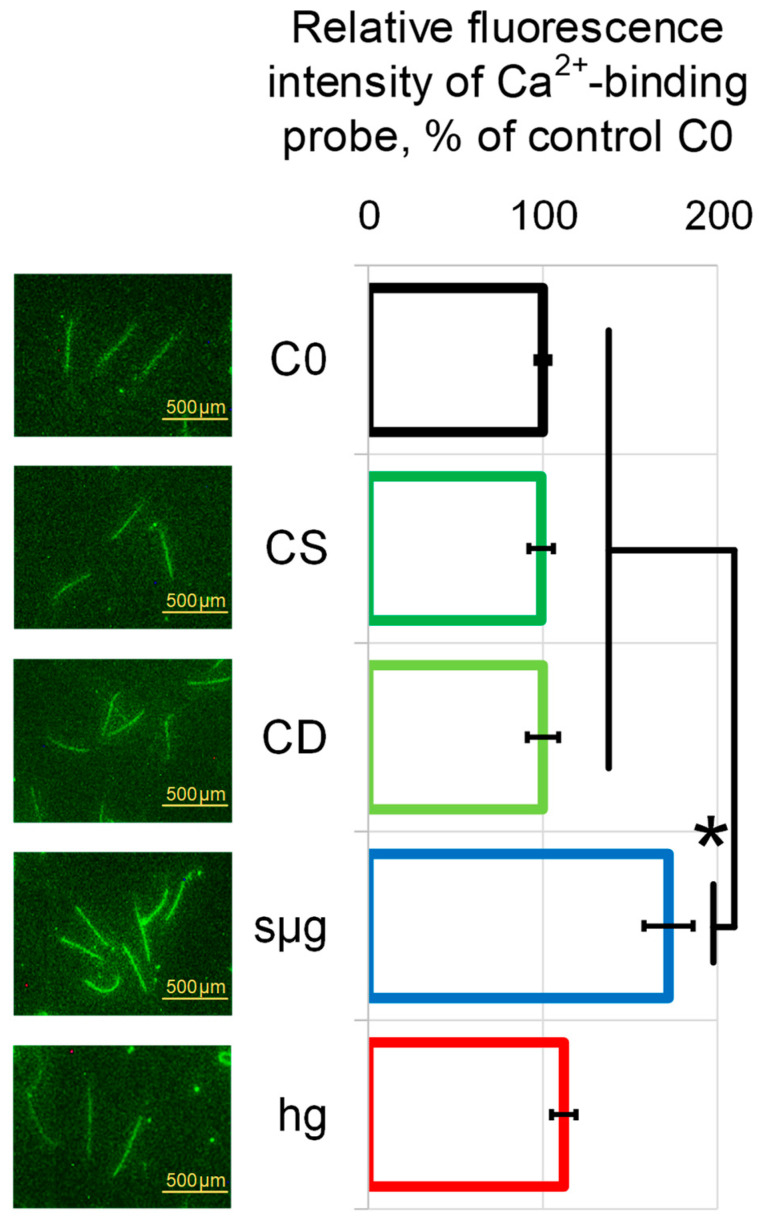
Relative calcium content in spermatozoa after 30 min exposure to simulated microgravity and 2 g hypergravity. Study groups—same as above, in Figure 1. Groups C0, CS, and CD did not differ from each other. *—*p* < 0.05 in comparison to CS. Typical images with bar 500 µm are shown on the left. Data are presented as mean ± SD of three independent experiments. In each independent experiment, at least 30 cells were assessed for fluorescence in each study group. Fluorescence in each study group was compared with CS group.

The relative content of free calcium in spermatozoa, which was visualized only in the neck and middle piece, as usual in the mammalian spermatozoa [20], did not differ between the control groups. In the simulated weightlessness group sµg, it was higher than the control level by 72% (*p* < 0.05), and in the hypergravity group, hg, there was no difference from the control (Figure 2).

### 2.3. Cytoskeletal Proteins Content

Among the proteins studied in the membrane and cytoplasmic fractions, there were no differences between the C0, CS, and CD groups.

**Figure 3:**
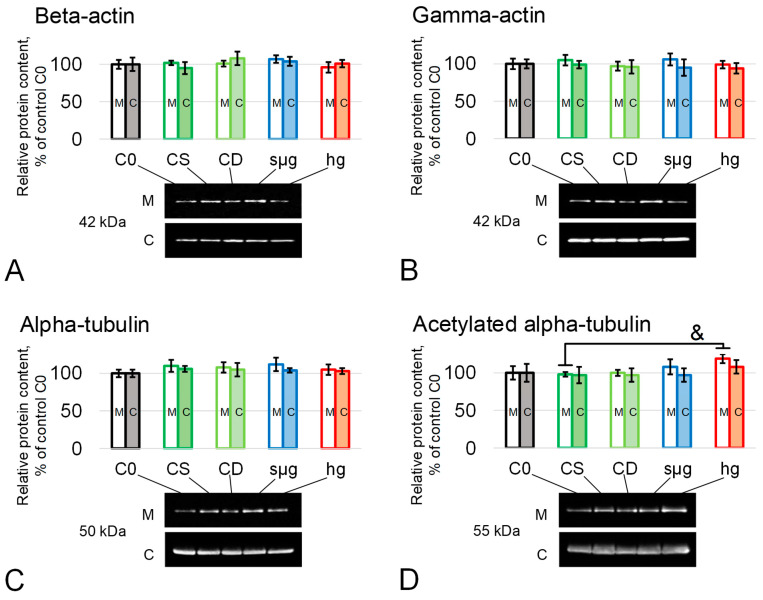
Relative content of the main proteins forming the sperm cytoskeleton after 30 min exposure to simulated micro- and hypergravity. Study groups—same as above, in Figure 1. M—membrane fraction, and C—cytoplasmic fraction. The protein content in groups C0, CS, and CD did not differ in either the membrane or cytoplasmic fractions. (**A**) Beta-actin, 42 kDa; (**B**) gamma-actin, 42 kDa; (**C**) alpha-tubulin, 50 kDa; and (**D**) acetylated alpha-tubulin, 55 kDa. &—*p* < 0.1 in comparison to CS. Typical Western blots are given under each histogram. Data are presented as mean ± SD of four biological replicas. Protein content in each study group was compared with the CS group.

Since actin is one of the main components of the submembrane cytoskeleton and acrosome of spermatozoa and tubulin forms the structural basis of the axoneme, their content in the studied groups was determined. Their relative content in the membrane and cytoplasmic fractions of spermatozoa after 30 min of exposure to simulated microgravity and hypergravity were generally not different from the control level (Figure 3A–D, Supplementary Materials). An exception was noted only for the relative content of acetylated tubulin, a marker of stable microtubules, in the membrane fraction in the hg group, where there was a trend towards an increase of 19% (*p* < 0.1) (Figure 3D, Supplementary Materials).

**Figure 4:**
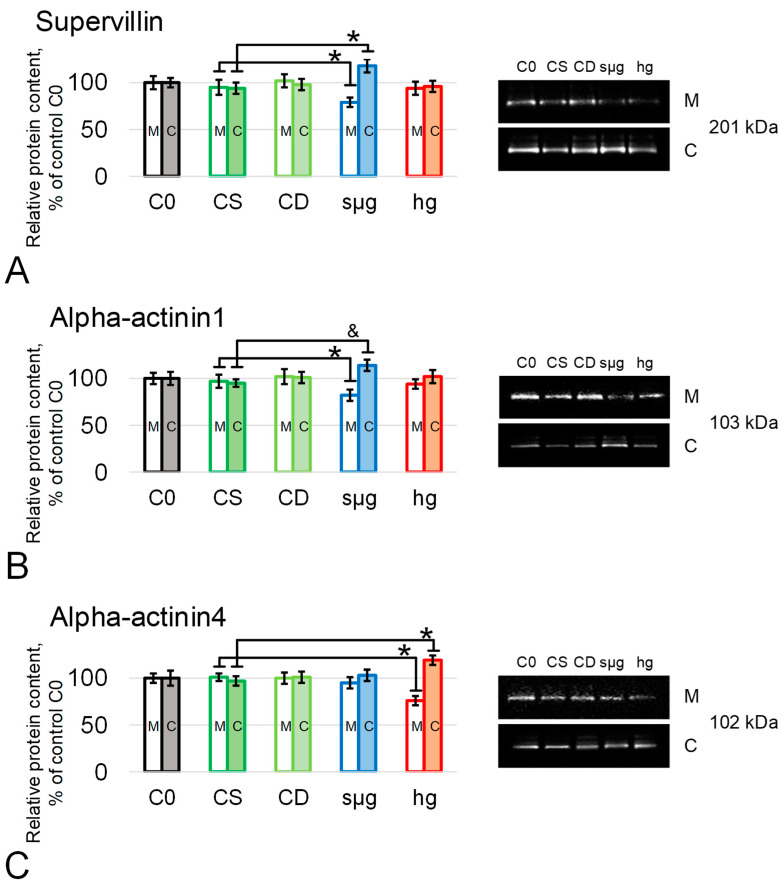
Relative content of actin-binding proteins in spermatozoa after 30 min exposure to simulated micro- and hypergravity. As above, study groups are the same as in Figure 1. M—membrane fraction, and C—cytoplasmic fraction. The protein content in groups C0, CS, and CD did not differ between themselves in either the membrane or cytoplasmic fractions. (**A**) Supervillin, 201 kDa; (**B**) alpha-actinin1, 103 kDa; and (**C**) alpha-actinin4, 102 kDa. *—*p* < 0.05, and &—*p* < 0.1 in comparison to CS. Typical Western blots are shown to the right of each histogram. Data are presented as mean ± SD of four biological replicas. Protein content in each study group was compared with the CS group.

Taking into account our previous data on the role of actin-binding proteins in cell mechanoreception [21,22], we determined their relative content, and redistribution between the membrane and cytoplasmic fractions was observed. Thus, the relative content of supervillin and alpha-actinin1 did not change in the hg group. But, in the sµg group, supervillin and alpha-actinin4 in the membrane fraction were lower than in the control by 21% and 18% (*p* < 0.05), respectively, and in the cytoplasmic fraction, they were higher by 18% (*p* < 0.1) and 14% (*p* < 0.05), respectively (Figure 4A,B, Supplementary Materials). The relative content of alpha-actinin4 in the sµg group did not differ from the control; in the hg group, in the membrane fraction it decreased by 24% (*p* < 0.05), and in the cytoplasmic fraction it increased by 19% (*p* < 0.05) compared to the corresponding control (Figure 4C, Supplementary Materials).

## 3. Discussion

The study of the mechanisms of interaction between the single cell and the gravitational field is still a pressing issue [21,22]. Primary acts of mechanoreception and subsequent mechanotransduction pathways remain poorly understood, although the search for sensors may lead to the development of fundamentally new preventive measures in the exploration of deep space.

Spermatozoa of most animal species function as single cells. Their motility is necessary for the delivery of male genetic material for the formation of a zygote. However, the signaling pathways for the development of an adaptive pattern of motor activity when the gravity force acting on spermatozoa changes, which have tubulin–dynein system of motility, remain insufficiently studied, although much more data have been obtained for the flagellar motility system [23].

The obtained results indicate that 30 min of exposure to altered gravity conditions lead to changes in the motility of mouse spermatozoa, moreover, differently under simulated micro- and hypergravity conditions, which may be of fundamental importance from the point of view of the analysis of mechanotransduction pathways. Under simulated weightlessness, sperm motility increased after 30 min (Figure 1A); although after an hour of exposure, it did not differ from the control and continued to fall further until 6 h, when it became lower than the control [13,14]. In other words, a fast (already in the first minutes of being in weightlessness [12]) transient increase in speed is a precursor to its decrease. Under the influence of 2 g hypergravity, motility decreases (Figure 1A) and continues to decrease with increasing exposure time [13].

Sperm motility is provided by the tubulin–dynein motor system localized in the tail part. Since the content of the main cytoskeletal proteins, which form the main structures—the acrosome and axoneme—remains intact (Figure 3), the change in motility can be mediated by regulatory phosphorylation/dephosphorylation of dynein [24,25,26,27,28,29,30,31,32]. It should be noted that phosphorylation by cAMP-dependent protein kinase A (PKA) or protein kinase C (PKC) activates motility [6,24,33,34], while by phosphatidylinositol 3-kinase (PI3K) or glycogen synthase kinase 3 (GSK3), it reduces the speed of movement [29,30].

We decided to try using 6-DMAP as a broad-spectrum kinase inhibitor, target inhibitors of PI3K and GSK3, as well as phosphatase inhibitors to determine their contribution to the motility-altering effect under simulated microgravity and hypergravity (Figure 1B–F).

The use of 6-DMAP did not change the speed in the control; after exposure to 2 g conditions, the speed remained reduced as well as without the addition of the inhibitor (Figure 1B). But, 6-DMAP prevented the effect of increasing the speed under simulated microgravity conditions (Figure 1B), which suggests the involvement of PKC and/or PKA in this process. However, both kinases carry out regulatory phosphorylation of dynein in a calcium-dependent manner; therefore, calcium ions are a necessary condition for ensuring sperm motility [20,35].

We determined the relative calcium content in spermatozoa after 30 min exposure to zero gravity and hypergravity. Indeed, after exposure to simulated microgravity, the relative calcium content in the cytoplasm of spermatozoa significantly increased compared to controls and hypergravity (Figure 2). A similar effect was shown for skeletal muscles [36] and already on the first day of unloading [37]. In muscle cells, we associated this effect with changes in the submembrane cytoskeleton, in particular, with the redistribution of Ca^2+^-dependent isoforms of the actin-binding protein alpha-actinin between the membrane and cytoplasmic fractions and possible activation of phospholipases [21].

Taken together, the mitigation of the speed increasing by the PKC inhibitor and the increase in calcium ions suggest activation of phospholipase C (PLC), which leads to increased hydrolysis of phosphatidylinositol 4,5-bisphosphate (PIP2). The resulting formation of inositol 1,4,5-trisphosphate (IP3) leads to an increase in calcium ions in the cytoplasm, and a second product of PIP2 hydrolysis, diacylglycerol (DAG), activates PKC.

Surprisingly, the same effect was caused by targeted inhibition of PI3K (Figure 1C). PI3K activity leads to accumulation of phosphatidylinositol 1,4,5-trisphosphate (PIP3) in the cell membrane as a result of PIP2 phosphorylation, which, accordingly, reduces the amount of substrates for PLC. However, it can be assumed that PI3K inhibition does not reduce the effects of microgravity but rather prevents their development. A decrease in PI3K activity leads to accumulation of PIP2, which is a cofactor of phospholipase D (PLD) [38]. In turn, PLD activates actin polymerization in a PI3K-dependent manner with the participation of PKA in mammalian spermatozoa [39,40,41], which can prevent the process of mechanotransduction and increasing sperm speed [22].

At the same time, inhibition of GSK3 led to an increase in the speed of sperm movement in all study groups, including the hypergravity group, but relative to the corresponding control, the speed in hg-group remained reduced (Figure 1D). However, inhibition of tyrosine phosphatase activity prevented a decrease in the speed of sperm movement under hypergravity conditions without affecting other study groups. The main tyrosine phosphatases are cytoplasmic and receptor-like transmembrane, associated with the submembrane cytoskeleton, among other things [42,43].

Interestingly, inhibition of Ser/Thr phosphatases by calyculin A prevented any changes in motility during 30 min exposure to altered gravity. Calyculin A increases the content of stress fibers in the cortical cytoskeleton [44,45], which in turn contributes to increased cell stiffness.

In our previous studies, we formulated a hypothesis and proposed a mechanism for the interaction of a single cell and a gravitational field. The cell exists at 1 g but can respond to both a decrease and an increase in gravity. A change in external mechanical stress, according to the laws of mechanics, will lead to a change in internal stress and, accordingly, to a deformation of the cortical cytoskeleton, which is present in all types of cells. We believe that in one case, this will be a stretching deformation and in the other, a compression deformation. Different deformations will lead to the migration of different proteins from the cortical cytoskeleton and, accordingly, to the launch of different signaling pathways and the formation of a different adaptation pattern [19,21,22,46].

Accordingly, the increase in the stiffness of the cortical cytoskeleton as a result of the action of calyculin A increases the resistance to deformation when the gravitational field acting on the cell changes, which can prevent the initiation of mechanotransduction, as we observed earlier [19] and in this case.

Connecting the mechanosensitivity of cells with the deformation of the cortical cytoskeleton and the migration of some binding proteins, in particular actin-binding proteins, into the cytoplasm [21,22], we determined the relative content of supervillin, alpha-actinin1, and alpha-actinin4 in the membrane and cytoplasmic fractions of spermatozoa after exposure to simulated micro- and hypergravity (Figure 4). Supervillin and alpha-actinin1 migrate from the membrane fraction to the cytoplasmic fraction under simulated microgravity (Figure 4A,B), while alpha-actinin4 does so under hypergravity (Figure 4C). Despite the high homology of alpha-actinin isoforms [47,48], they are localized in different areas of the cytoskeleton: alpha-actinin4 anchors the cortical cytoskeleton to the membrane and forms interactions with other proteins, while alpha-actinin1 is localized along stress fibers and microfilament bundles [49,50], as is supervillin [51,52]. Thus, in mouse spermatozoa, depending on the transition to either simulated micro- or hypergravity, either alpha-actinin1 and supervillin (form bundles) or alpha-actinin4 (forms networks) migrate from the cortical cytoskeleton. These data may confirm the previously proposed mechanism of interaction between the cell and the gravitational field and correlate with similar previous results [19,21,22,46].

Migration of different proteins from the cortical cytoskeleton can trigger different mechanotransduction pathways and form a corresponding adaptation pattern [20,21]. It is well known that alpha-actinin1 interacts with phospholipase D in cardiomyocytes and inhibits its activity [53], but apparently there is a similar relationship with phospholipase C [54]. A decrease in the content of alpha-actinin1 in the cortical cytoskeleton increases the activity of phospholipases, leading to PIP2 hydrolysis, an increase in calcium content, and activation of protein kinase C, which then lead to the observed increase in sperm motility under simulated weightlessness. On the other hand, in spermatozoa, alpha-actinin interacts with tyrosine phosphatase [55]. Under hypergravity conditions, the migration of alpha-actinin4, which is a substrate of the tyrosine phosphatase PTP1B [56], can cause its activation and indirectly reduce sperm motility.

## 4. Materials and Methods

### 4.1. Experimental Design

The study was conducted on spermatozoa of Balb/c mice. The animals were euthanized by inhalation anesthesia; then, the epididymis and duct deferens were isolated and placed in α-MEM with 10% bovine serum. Next, the tissues were crushed and incubated on a shaker for 40 min at a temperature of +37 °C. Then, the resulting suspension was passed through a filter with a hole diameter of 70 μm, and thus spermatozoa were obtained, which were divided into the following study groups:

- C0—control at 0 min of exposure—intact spermatozoa just after collection;
- CS—control static—intact spermatozoa after 30 min of exposure without any impacts;
- CD—control dynamic—spermatozoa after 30 min of exposure on shaker with similar velocity of rotation in one plane;
- sµg—simulated microgravity during 30 min;
- hg—hypergravity at 2 g level during 30 min.

All tubes with spermatozoa were completely filled with the medium α-MEM with 10% bovine serum (up to 200 µL pure or with any inhibitors when determining the speed of sperm movement, depending on the series of experiments) to avoid the majority of artifacts associated with the movement of liquid layers. The tubes with spermatozoa were hermetically sealed, and concentration was 10^7^ cells/mL. Simulated microgravity conditions were created using a 3D-clinostat, and hypergravity conditions were created by centrifugation, using in both cases the Gravity Controller Gravite (Gravite^®^, GC-US-RCE010001, Space Bio-Laboratories Co., Ltd., Hiroshima, Japan). In each round of the experiment, the test tube containing sperm was placed exactly in the center of the platform, on the axis of rotation. The exposure duration was 30 min at +37 °C, and controls were at the same conditions. To simulate microgravity, we used a mode with the constant velocity in which the superposition of the gravity vector acting on a test tube placed in the center of the platform is equal to zero on average over 15 s. All measurements were performed within 1 min after removing the tube with spermatozoa from the device without stopping it to avoid any additional artifacts.

After exposure, depending on the series of experiments,

- Spermatozoa were immediately transferred to the Makler chamber and the speed of movement was determined;
- A smear was made on glass to determine the relative calcium content;
- Frozen for subsequent protein extraction.

To determine the significance of experimental data, at least three biological replicates were used. All the manipulations with animals and experimental procedures were approved by the Commission on Biomedical Ethics of the SSC RF Institute of Biomedical Problems RAS (Protocol #521, 25 September 2019).

### 4.2. Measuring Sperm Motility

To determine the role of protein kinases (PKs) and protein phosphatases (PPs) in changing sperm motility, several series of experiments were conducted that differed in the incubation medium:

- No inhibitors were added to the incubation medium;
- Medium with the addition of a broad-spectrum protein kinase inhibitor—0.5 mM 6-(dimethylamino) purine (6-DMAP) (ab145307, Abcam, Cambridge, UK) [57];
- Medium with the target inhibitor of phosphatidylinositol-3-kinase (PI3K)—20 μM wortmannin (CAS 19545-26-7, Calbiochem, Merck, San-Diego, CA, USA) [58];
- Medium with the selective inhibitor of glycogen synthase kinase 3 (GSK3)—1 μM AR-A014418 (A3230, Sigma Aldrich, Merck, Burlington, MA, USA) [59,60];
- Medium with the inhibitor of tyrosine phosphatase (Tyr PP)—200 μM sodium orthovanadate Na3VO4 (CAS 13721-39-6, Calbiochem, San Diego, CA, USA) [42,61];
- Medium with the inhibitor of serine/threonine phosphatase (Ser/Thr PP)—20 nM calyculin A (# 19-139, Millipore, Merck, St. Luis, MO, USA) [62].

As previously described [13], after exposure, 5 μL of sperm suspension from each study group was placed in a Makler chamber (Sefi Medical Instruments Ltd., Haifa, Israel) and placed under a phase-contrast objective (total magnification was 200×) of a microscope (Eclipse E200 MV, Nikon, Tokyo, Japan) equipped with a Basler puA1600-60uc color camera with e2V EV76C570 CMOS sensor and 60 frames per second with 2 megapixel resolution (Basler AG, Ahrensburg, Germany). Video of sperm movement was recorded and analyzed using the plugin Manual Tracking in Fiji software for Windows (64-bit) (open access https://fiji.sc, date of access 11 June 2024). Speed, in µm/s, of spermatozoa was calculated as distance traveled by head per second.

### 4.3. Determination of Relative Calcium Content

To obtain data on the change in the relative content of free calcium in the cytoplasm of spermatozoa, immediately after exposure a drop of the suspension was applied to a glass slide and a smear was made. It was then dried and fixed in a solution containing equal parts of phosphate-buffered saline and 4% buffered formalin for 30 min at +37 °C with gentle shaking.

Next, the preparation was washed and incubated in the dark at room temperature with 2 µM Fluo4-AM (F14201, Molecular Probes, Invitrogen, Eugene, OR, USA)—a fluorescent calcium-binding probe capable of penetrating the cell membrane. After washing, fluorescence was visualized using an IX73 inverted fluorescent microscope (Olympus Corporation, Tokyo, Japan) with 40× magnification. Images were processed using ImageJ in Fiji software for Windows (64-bit) (open access https://imagej.net/software/fiji/, date of access 18 July 2024).

### 4.4. Western Blotting

The membrane and cytoplasmic fraction of proteins were isolated from frozen spermatozoa after exposure according to the protocol [63]. Then, the concentration was measured, and, accordingly, the equal amount of protein was applied to each well of the gel, electrophoresis was performed, followed by transfer to a nitrocellulose membrane, then staining with Ponceau S (for control transfer and normalization) and staining with specific primary antibodies. Antibodies to beta-actin, gamma-actin, acetylated alpha-tubulin, alpha-actinin4, supervillin (#sc-81178, #sc-65638, #sc-23950, #sc-393495 and #sc-53556, accordingly, Santa Cruz Biotechnology, Inc., Santa Cruz, CA, USA), alpha-tubulin, and alpha-actinin1 (#ab52866 and #ab50599, accordingly, Abcam, Cambridge, UK) were used at the dilutions recommended by the manufacturers. HRP-linked antibodies to detect rat Ig, mouse IgG, and rabbit IgG (#7077S, #7076S, and #7074S, accordingly, Cell Signaling Technology, Inc., Danvers, MA, USA) were used as secondary antibodies. The membranes were then treated with SuperSignal™ West Femto Maximum Sensitivity Substrate (#34096, Thermo Scientific™, Waltham, MA, USA), and the protein bands were revealed using ChemiDoc XR+ System and processed using Image Lab 6.1 Software for Windows (Bio-Rad Laboratories, Hercules, CA, USA).

### 4.5. Statistical Analysis

Three independent experiments were performed to evaluate sperm motility and calcium content. In each of these experiments, the motility of at least 50 cells and the fluorescence of at least 30 cells were determined in each study group. Four independent experiments were performed to determine the relative protein content. In each experiment, the data were compared with the CS group using a *t*-test to assess statistical differences in these two groups. Experimental data are presented as the mean values obtained in independent experiments ± its standard deviation (M ± SD). To assess the differences in means between each experimental group and the CS control group, one-way ANOVA with post hoc Tukey test was used with a significance level of *p* < 0.05, and data were processed using Origin 2019 software.

## 5. Conclusions

**Figure 5:**
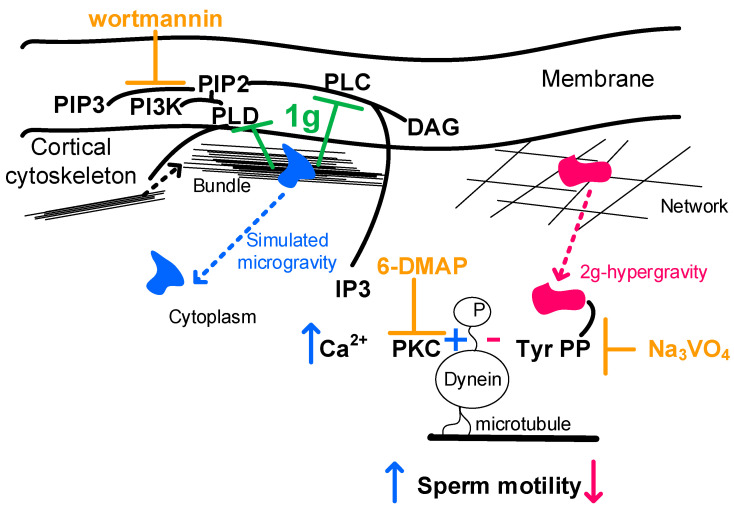
Possible scheme of events of formation of pattern of sperm motility after 30 min exposure to conditions of simulated weightlessness and 2 g hypergravity. Detailed description is given in the text. Proteins forming bundles of filaments—migrate into cytoplasm under simulated microgravity—are shown in blue. Proteins forming networks of filaments—migrate into cytoplasm under hypergravity—are shown in red. State under normal gravity is shown in green. Plus sign denotes activating effect, and minus sign denotes suppressive effect. Up arrows denote observed increase in parameter, and down arrows denote decrease. PIP2—phosphatidylinositol 4,5-bisphosphate, PIP3—phosphatidylinositol 1,4,5-trisphosphate, IP3—inositol 1,4,5-trisphosphate, DAG—diacylglycerol, PLC—phospholipase C, PI3K—phosphatidylinositol 3-kinase, PKC—protein kinase C, and Tyr PP—tyrosine phosphatase. Inhibitors are highlighted in yellow: 6-DMAP, 6-(dimethylamino) purine, inhibits PKC; wortmannin inhibits PI3K; and Na_3_VO_4_, sodium orthovanadate, inhibits Tyr PP.

Summarizing the obtained results and the above, the following sequence of events can be proposed (Figure 5).

Under simulated microgravity conditions (Figure 5, left part), proteins that bind filament bundles and stress fibers, in particular alpha-actinin1, migrate from the cortical cytoskeleton, which may lead to an increase in phospholipase activity. On the one hand, phospholipase C hydrolyzes PIP2, and calcium content and PKC activity increase, which lead to a rapid effect of increasing sperm motility. However, a simultaneous increase in phospholipase D activity leads to PI3K activation, a shift in the PIP2/PIP3 balance, and over time, a decrease in sperm motility develops, which we observed with longer exposure. However, in the early stages of microgravity exposure, PI3K inhibition allows maintaining the PIP2 pool, which allows phospholipase D to stimulate F-actin formation and increase cellular resistance to mechanical stress, similar to calyculin A.

Under hypergravity conditions (Figure 5, right side), alpha-actinin4, which forms the cytoskeleton–membrane interaction and organizes the filament network in the cortical cytoskeleton, migrates into the cytoplasm and the activity of its possible phosphatase PTP1B increases, which leads to dynein dephosphorylation and the observed decrease in the speed of movement. Accordingly, this decrease is prevented by the tyrosine phosphatase inhibitor sodium orthovanadate.

It should be noted that the presented scheme is, in many respects, hypothetical, which is associated with the complex mutual influence of all participants in the signal cascades and the constantly emerging new ways of their interaction. However, in general, the obtained results allow us to assume that even a short incubation of spermatozoa under altered gravity, for example, immediately after deconservation in extraterrestrial conditions, leads to a change in their speed of movement and the launch of mechanotransduction pathways.

The motor activity of spermatozoa is a necessary condition for the natural maintenance of the species, therefore, the analysis of the mechanisms of its change both in zero gravity and under the influence of overloads, in the future, may be important for space medicine and help to provide new approaches in the artificial reproductive technologies.

### Limitations of the Study

The main limitation of this study is related to the method of weightlessness simulation used, which we created by Gravity Controller Gravite (Gravite^®^, GC-US-RCE010001, Space Bio-Laboratories Co., Ltd., Hiroshima, Japan) based on the principle of 3D-clinostat. This method has a number of limitations related to the formation of shear stress [64]. However, we tried to minimize the influence of this fact on the obtained results. On the one hand, in this device, the direction of rotation is random, while the speed remains constant, respectively, there is no acceleration. On the other hand, the measured parameters in the dynamic control group did not differ from the static one. Finally, the changes in simulated weightlessness and hypergravity were different, although possible shear stress was present in both cases. In addition, this device is also used to study other types of cells, even attached cultures [65]. However, it should be noted that this is a model experiment but not real weightlessness, which is a limitation of this study.

Another limitation of the study is related to the use of sodium orthovanadate as a well-known inhibitor of tyrosine phosphatases. At the same time, at a concentration of 40 nM, it inhibits P-type ATPases with 50% efficiency, which play an important role in the maturation and formation of the acrosome and the acrosome reaction [66].

Finally, the Western blot is a semi-quantitative method, and since we detected not very large changes in protein content in different fractions, as is often the case under the influence of micro- and hypergravity at the 2 g level, this requires additional verification in other approaches and is a limitation of this study.

## Data Availability

All data generated or analyzed during this study are included in this article.

## Supplementary Materials

The following supporting information can be downloaded at: https://www.mdpi.com/article/10.3390/ijms252413561/s1.
