# Supplementary figures and images for: The Motility of Mouse Spermatozoa Changes Differentially After 30-Minute Exposure Under Simulating Weightlessness and Hypergravity
Source: Int J Mol Sci. 2024 Dec 18;25(24):13561. doi: 10.3390/ijms252413561 (PMC11678010; doi:10.3390/ijms252413561)

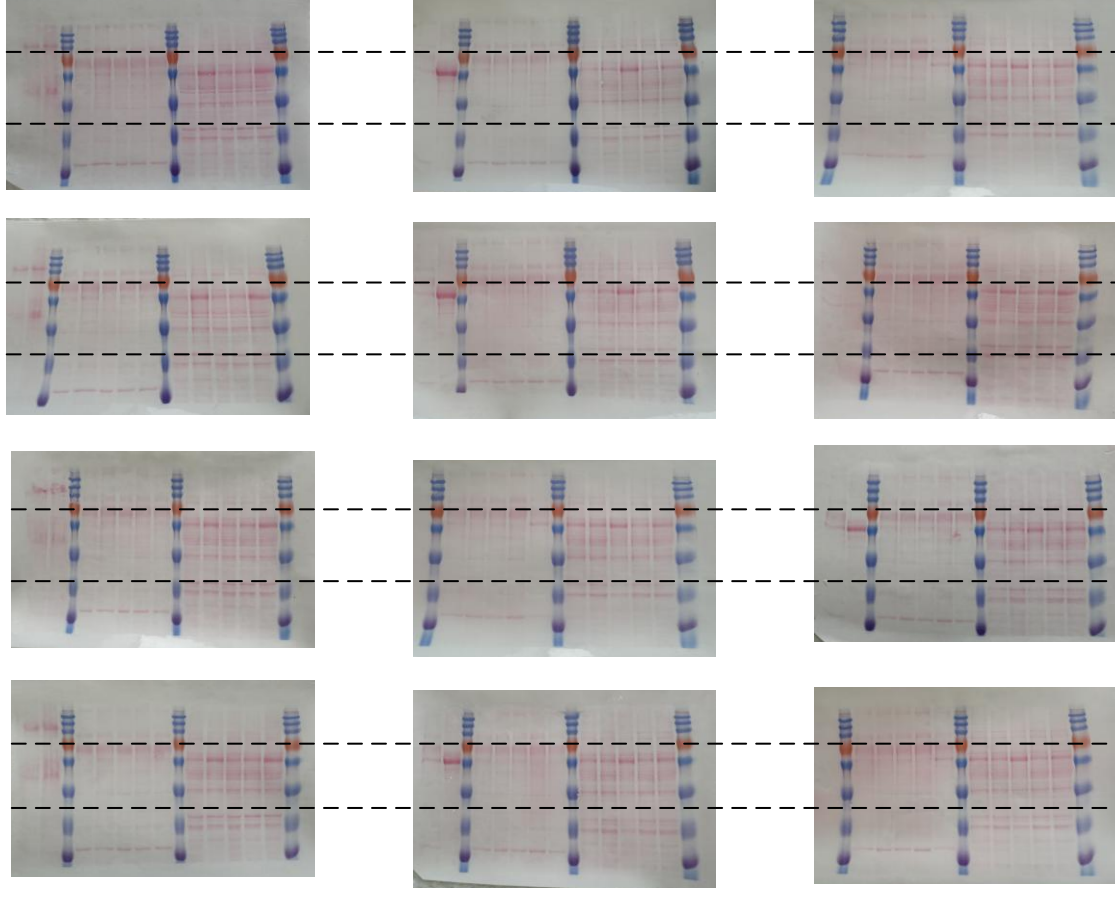

Supplement: Supplementary file 1 [file ijms-25-13561-s001.zip › ijms-3362260-supplementary.pdf]
